# The Shear Bond Strength between Milled Denture Base Materials and Artificial Teeth: A Systematic Review

**DOI:** 10.3390/dj11030066

**Published:** 2023-03-01

**Authors:** Vladimir Prpic, Amir Catic, Sonja Kraljevic Simunkovic, Lana Bergman, Samir Cimic

**Affiliations:** 1 Department of Fixed Prosthodontics, School of Dental Medicine, University of Zagreb, 10000 Zagreb, Croatia; 2 Department of Prosthodontics, University Hospital Centre Zagreb, 10000 Zagreb, Croatia; 3 Department of Removable Prosthodontics, School of Dental Medicine, University of Zagreb, 10000 Zagreb, Croatia

**Keywords:** dentures, CAD/CAM, artificial teeth, PRISMA 2020

## Abstract

The data about bond strength between digitally produced denture base resins and artificial teeth are scarce. Several studies investigated shear bond strength values of milled denture base resins and different types of artificial teeth. The purpose of the present study was to compare and evaluate the available evidence through a systematic review. A bibliographic search was conducted in PubMed, Scopus, and Web of Science to assess adequate studies published up to 1 June 2022. This review followed the PRISMA (Preferred Reporting Items for Systematic Reviews and Meta-Analyses) guidelines. The appropriate studies that determined the shear bond strength values between milled denture base resins and artificial teeth were selected. The initial search identified 103 studies, which were included in the PRISMA 2020 flow diagram for new systematic reviews. Three studies met the inclusion criteria, and all of them present a moderate risk of bias (score 6). Two studies found no statistical differences between heat-polymerized and CAD/CAM (milled) denture base materials when attached with different types of artificial teeth, while one study showed higher values of CAD/CAM (milled) denture base materials. Bonding agents ensure bonding strength at least similar to the conventional methods. In order to improve the quality of future studies, it would be advantageous to use a larger number of specimens with standardized dimensions and a blinded testing machine operator to decrease the risk of bias.

## 1. Introduction

Edentulism is defined as a complete absence of teeth and is considered a disability by the World Health Organization (WHO) [1]. The prevalence of reported causes for tooth loss is as follows: caries (36.0% to 55.3%); periodontitis (24.8% to 38.1%); trauma (0.8% to 4.4%), periapical disease (7.3% to 19.1%); orthodontics (2.5% to 7.2%); and other reasons (4.5% to 9.2%) [2]. Following tooth loss, the underlying bone continues to resorb, and there are changes to patients’ facial appearance and facial height [3]. Moreover, elderly people who wear complete dentures are more likely to experience denture stomatitis, an inflammatory condition of the palatal mucosa, as well as angular cheilitis, oral candidosis, and traumatic ulcers [3]. Dental health has a significant impact on quality of life; edentulism negatively affects patients’ quality of life because it decreases chewing capacity, enervates phonetics, and impairs aesthetics [4]. The loss of chewing efficacy can result in a decreased intake of food (e.g., vegetables and fruit), which could lead to malnutrition. Simultaneously, edentulous patients cannot meet dietary recommendations [5]. In addition, edentulism can be connected with several systematic conditions such as pneumonia, adiposity, neck and head carcinoma, and a higher risk of mortality [1]. Studies showed different prevalences of edentulism. Females have higher prevalence of edentulism when compared to males, especially females with poor profit and lower degree of education [6]. In a review, Polzer et al. [7] determined that the prevalence of edentulism ranges from 1.3% to 78% for people aged 65 and older. Inter- and intracountry differences in the prevalence of edentulism exist, but comparison between nations is challenging because of different factors (e.g., erudition, economical instance, dental health competence, and beliefs and attitudes towards oral health) which can influence the outcome [3,8]. Manifestations of edentulism are constantly falling in developed countries, whilst in developing countries, the opposite trend has been observed [9]. Nevertheless, a study by Douglass et al. [10] showed increasing edentulism as a result of aging and longer life expectancy. The higher number of elderly people could be caused by demographic changes which can be seen in the majority of countries [11]. A total of 703 million people worldwide are aged 65 years and above, and it is predicted that the given number will double by 2050. The population of people aged 80 years and above is 143 million, which, following the current trend, is likely to triple by 2050 [11]. Consequently, the necessity of treating patients suffering from edentulism will be greater than ever.

Complete dentures have been used for many years and are considered the gold standard for treating patients with this condition [4,7,12,13,14]. Although other types of prosthodontic solutions for edentulism are accessible, such as implant-supported dentures, most patients decide to wear conventional ones, mainly because of financial causes [15,16,17]. Until today, polymethyl methacrylate (PMMA) has remained the most accepted denture base resin [18,19]. PMMA conforms to some of the requirements for a perfect denture base resin, which include easy mending, optimal appearance, and a tolerable price [13,20]. Common manipulation is also one of the advantages and is started by mixing methyl methacrylate (monomer) and polymethyl methacrylate (polymer) [18]. The polymerization process of a given type of PMMA (heat-polymerized resin) is terminated after a certain exposure time to heat. Despite all the advantages, polymerization shrinkage of 6% can be interpreted as the main disadvantage [18]. Moreover, the chemical reaction between methyl methacrylate and polymethyl methacrylate is never complete. Leftovers of the residual monomer after the polymerization process can modify the mechanical properties of the material and cause allergic reactions (e.g., oedema, stomatitis, and ulcerations) [18,21].

Bonding between the denture base material and the artificial teeth is imperative for completeness of dentures and patient’s quality of life. Resin artificial teeth are used more frequently when compared to porcelain ones because of the chemical bonding that occurs and simple occlusal adaptation [22]. Furthermore, resin denture teeth are persistent to thermal changes and are less pervious to fracture under impact [22].

Debonding of an artificial tooth from a denture base is a frequent clinical situation, and, according to some studies, 30% of all denture adjustments are due to unsuccessful bonding, often in the anterior parts of dentures [23,24,25,26]. The main reasons for detachment between a denture base and denture teeth include the presence of wax on the ridge lap surface of an artificial tooth, an inattentive application of the separating agent, and the polymerization technique used for the fabrication of denture base resin [27,28]. Beside chemical and mechanical modifications of the ridge lap surface, prefabricated teeth with different composition (acrylic/composite) can be expected to have differences in bond strength values between teeth and resin [29].

Recently, with developments in science and technology, new materials in removable prosthodontics have been disclosed [30]. Technological progress in dental medicine has enabled the usage of digital methods (computer-aided design/computer-aided manufacturing [CAD/CAM]), including subtractive and additive technologies for denture base manufacturing [29,31,32,33]. Digital methods allow the fabrication of a denture base in one piece and ensure the option to adhere artificial teeth with adequate adhesive [14].

Presently, two fabrication methods are attainable when using the milling method: (a) production of a denture base and denture teeth from one portion, and (b) production of a denture base and denture teeth as segregate portions, requiring a bonding procedure [29,34,35,36]. Currently, the second method is more popular for fabricating dentures [34,35,37,38,39], mainly due to better fitting and retention [36,38,40] and utilization of the commercially affordable denture teeth with enhanced physical and aesthetic properties [41,42]. The pros of the above-mentioned digital methods are quicker denture manufacturing with a lower number of phases in the workflow that can decrease the possibility of errors [14]. When using a conventional workflow, the bonding between denture base resin and denture tooth resin eventuates during the polymerization process of denture base acrylics while the artificial teeth are in mould [43]. In a digital workflow, the bonding of a denture base and a denture tooth resin must be conducted apart, which requires bonding material or poly(methyl methacrylate) (PMMA) appliance in a powder/liquid form (as in the conventional manner) [43].

To the authors’ knowledge, there has not been a systematic review of studies that compared bond strength values between CAD/CAM (milled) denture base resins and artificial teeth. Specifically, the ridge lap surface of artificial teeth tested in the accessible studies should have been intact to fulfil the inclusion criteria for the present review. The aim of the present systematic review was to compare shear bond strength values between elected studies, to enable the selection of optimal material for a given clinical situation, and to seek the answer for the question: are the shear bond strength values of CAD/CAM (milled) and conventional denture base resins comparable when attached with different types of artificial teeth?

## 2. Materials and Methods

The present systematic review was conducted in accordance with PRISMA 2020 (Preferred Reporting Items for Systematic Reviews and Meta-Analyses) guidelines [44].

Studies that examined and compared the shear bond strength between CAD/CAM (milled) denture base resins and artificial teeth with control groups (heat-polymerized denture base resin) were included in the review. The exclusion criteria comprised case reports, non-full text studies (e.g., abstracts), case series, editorials, and interviews. The PICO (Population/ Problem, Intervention, Control or Comparison, and Outcome) framework was used to develop systematic review research questions, such as PRISMA 2020, [44] recommends. The PICO question was: is the shear bond strength (SBS) between CAD/CAM (milled) denture base resins and artificial teeth comparable with the shear bond strength between conventional heat-polymerized denture base resins and artificial teeth? To answer the given question, a search strategy was performed with keywords by means of every part of the PICO question.

**Figure 1:**
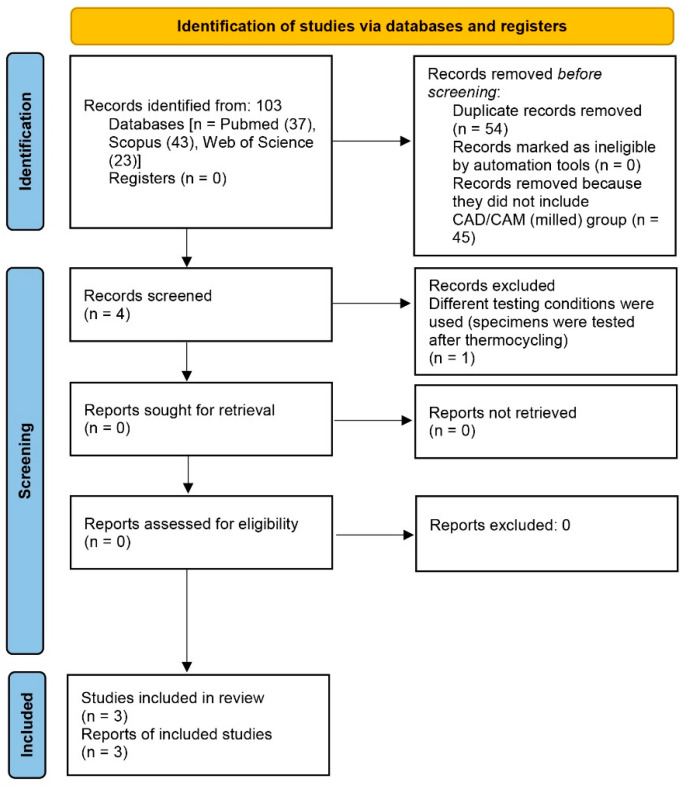
PRISMA 2020 flow diagram for new systematic reviews.

Detailed bibliographic research was conducted in the following databases: PubMed, Scopus, and Web of Science to assess adequate studies published up to 1 June 2022. Inclusion criteria encompassed full-text in vitro studies written in English and collating shear bond strength between CAD/CAM (milled) and heat-polymerized denture base resins using defined specifications during the testing procedure. Studies that did not meet the given criteria were excluded, as were registers, websites, organizations, and reference lists. The search keywords were CAD/CAM (milled) denture base resins, PMMA, shear bond strength, CAD/CAM, complete dentures, and artificial teeth. The search strategy was accomplished with keywords separated by the Boolean operator AND. The search used the following: shear bond strength AND denture base resins AND artificial teeth. A total number of studies that were found is presented in Figure 1.

Two skilled reviewers (S.C. and V.P.) evaluated the titles of all attained studies in accordance with the inclusion criteria. Afterwards, abstracts of the chosen studies were previewed, and the ones of interest were selected for full-text inquiry. The data from selected and reviewed studies were divided into a table. Both reviewers should have agreed on certain studies to be selected for analysis. Third and fourth reviewers (A.C. and S.K.S.) were consulted when necessary. The data summary protocol includes the following: the information on the authors/year, title of the study, type of the study, number of specimens, investigated properties, composition of tested materials, results, and conclusions (Table 1).

The evaluation of the risk of bias in the included studies in the present review was analyzed with the following modifiers: (1) samples obtained through a standardized process, (2) single operator of the machine, (3) sample size calculation, (4) blinding of the testing machine operator, and (5) specimens, tests, and formulas according to standard specifications (Table 2).

The score was 0 if the study clearly reported the specific parameter, 1 if the study insufficiently or unclearly reported the specific parameter, and 2 if it was not possible to detect the certain data in the study. Studies that scored between 0 and 3 were categorized as having a low risk of bias, studies with scores between 4 and 7 as moderate-risk, and scores between 8 and 10 as high-risk (Table 2).

## 3. Results

Initially, one hundred and three studies (*n* = 103) were found during the database search. After the screening procedure, studies that used other tests than the shear bond strength test (e.g., flexural bond strength), studies that evaluated the shear bond strength of 3D printed denture base resins, and duplicates were excluded by using the PRISMA 2020 flow diagram for new systematic reviews (Figure 1). Altogether, three studies met the inclusion criteria and were further analyzed: “Comparative Effect of Different Surface Treatments on the Shear Bond Strength of Two Types of Artificial Teeth Bonded to Two Types of Denture Base Resins” by Helal et al. [4], “Comparison of shear bond strengths of different types of denture teeth to different denture base resins” by Prpić et al. [29], and “Shear bond strength between CAD/CAM denture base resin and denture artificial teeth when bonded with resin cement” by Han et al. [45]. Table 1 presents a summary of the data extracted data from the included investigations.

All afore-mentioned studies [4,29,45] show a moderate risk of bias (score 6). The scores for the following modifiers: single operator of the machine, depiction of the sample size calculation, and blinding of the testing machine operator were generally low (score 2) when compared with other risk of bias modifiers (samples obtained through a standardized process and specimens, tests, and formulas according to standard specifications) (score 0). Table 2 shows a risk of bias for the included studies.

The highest shear bond strength values (21.80 ± 3.00 MPa) between CAD/CAM (milled) denture base resin and artificial teeth were found in the study by Han et al. [45], while the lowest (7.92 ± 0.61 MPa) were measured in the study by Helal et al. [4]. The highest shear bond strength values (19.31 ± 5.16 MPa) between heat-polymerized resin and artificial teeth were determined in a study by Han et al. [45], while the lowest (3.28 ± 0.92 MPa) were measured in the study by Helal et al. [4]. The given results of shear bond strength between milled CAD/CAM material and artificial teeth compared to heat-polymerized resin were similar [29,45] or higher [4] (Table 1).

## 4. Discussion

The effects of complete tooth loss can be minimized through rehabilitation with dental prostheses, which is the most cost-effective and most widely used treatment. Modifications in the orofacial muscles, combined with the necessary prosthodontic rehabilitation, repair impaired self-esteem and improve confidence by renewing the patient’s appearance [46].

Shear bond strength is the strength between two materials, and it shows how much each material resists the load before it fractures under a shear force [29,47]. Considering the fact that debonding can occur between denture base resins and an artificial tooth for different reasons, shear bond strength values should be as high as possible [29]. Generally, the shear bond strength test is standardly used for investigating the bond strength between denture base resin and artificial tooth resin [4,29,45,48,49,50]. Apart from the shear bond strength testing, some other testing procedures are available. Flexural bond strength (FBS) testing represents a novel method for the measurement of bond strength [51]. However, not enough studies have been conducted so far regarding flexural bond strength as a new testing modality [51]. Shear bond strength testing is most widely used because of its simplicity and ease of specimen preparation—no additional treatment of specimens is required after the bonding proceeding [52,53]. Considering the fact that the crosshead speed of the universal testing machine has an impact on shear bond strength values, the recommended crosshead speed is set between 0.45 and 1.05 mm/min [43], which all included studies accomplished.

The reported values of shear bond strength between denture base resins and artificial teeth vary [48,49,54,55,56,57]. This could be credited to the absence of standardization of testing methods as well as the variety of denture base materials available on the market [29]. The literature agrees that with different types of denture base materials (cold-polymerized, heat-polymerized, microwave-polymerized, light-polymerized, and others) differences in shear bond strength values to prefabricated denture teeth can be expected [48,55]. Studies also showed that different types/materials of prefabricated denture teeth can have different shear bond strengths with denture base resin. When comparing monomer diffusion between heat-polymerized denture base resin and different types of artificial teeth during the bonding procedure, it could be noted that the diffusion rate is higher in acrylic teeth in comparison with cross-linked and composite teeth [45]. Since acrylic teeth can chemically bond to heat-polymerized denture base resin via high monomer diffusion, high shear bond strength values are foreseen [29,58]. To achieve adequate chemical bonding, as with a milled denture base, different bonding agents are applied [29,59,60,61,62]. In a systematic review of bonding to CAD/CAM indirect resin materials, Mine et al. [62] concluded that the appliance of methyl methacrylate adhesives enhances the bonding of CAD/CAM (milled) materials.

A review of recent studies (Table 1) showed different results for the shear bond strength between CAD/CAM (milled) denture base resins and artificial teeth and control groups (heat-polymerized denture base resins and artificial teeth). In a study by Prpić et al. [29] and a study by Han et al. [45], shear bond strength values between (different) denture teeth and CAD/CAM (milled) denture base resin had comparable results to the same teeth connected with heat-polymerized denture base resin. Prpić et al. [29] compared the shear bond strength values of different types of artificial teeth (acrylic, nanohybrid composite, and cross-linked) attached to CAD/CAM (milled) denture base resin. Similarly, Han et al. [45] examined shear bond strength values between CAD/CAM (milled) denture base resins and composites with fillers and cross-linked teeth. Both studies used bonding agents for attaching teeth to milled denture base material and had a control group with heat-polymerized resin. Contrary to these studies by Prpić et al. [29] and Han et al. [45], Helal et al. [4] (Table 1) reported higher shear bond strength values between CAD/CAM (milled) denture base resin and two types of denture teeth (acrylic and composite) when compared to conventional heat-polymerized acrylics. This can be interpreted by the volumetric shrinkage reported in heat-polymerized acrylics, which could decrease shear bond strength values [4]. Still, the determined results of studies (Table 1) agree that the use of a bonding agent is at least comparable to the conventional method (compression moulding technique).

As follows from the previous paragraph, two studies [29,45] indicated that no statistical differences were observed between heat-polymerized and CAD/CAM (milled) denture base materials when attached to different types of artificial teeth, and one study showed higher values for CAD/CAM (milled) denture base materials [4]. Conventional hot water/bath polymerization represents the most effective and eligible procedure for denture fabrication as well as for bonding artificial teeth to denture base material [63]. Considering this fact, shear bond strength values between CAD/CAM (milled) denture base materials and denture teeth comparable to the conventional method can be considered a success. In other words, currently available bonding agents used to achieve optimal bonding between CAD/CAM (milled) denture base materials and artificial teeth demonstrate high shear bond strength values, equivalent to the bonding values between conventional denture base materials and artificial teeth [29].

Traditional fabrication techniques for removable dental prostheses are well known and are still frequently used in clinical practice today. These traditional complete denture protocols require multiple patient visits as well as extensive chairside and laboratory time [64]. Recent advancements have made it possible to incorporate computer-aided design and manufacturing (CAD/CAM) technologies into the complete denture manufacturing process. The constant evolution and improvement of technology has resulted in an exponential increase in the number of providers and systems available on the market today [64]. Some studies indicate that complete dentures produced using CAD/CAM (milled) technologies are as accurate as, or better than, conventionally produced dentures and have better material properties [38,65,66]. Moreover, high levels of patient satisfaction with digitally produced dentures have been reported [40,67]. To conclude, stomatognathic system harmony and overall health can efficiently be re-established with complete dentures, [46] and it is expected that digital technologies will provide even more efficient therapy in the future.

Presently, there are no generally acknowledged guidelines for estimating the quality of in vitro studies with a risk of bias assessment. Hence, the risk of bias was calculated according to some previously published studies [68,69]. Since three studies were included, a meta-analysis could not be performed, which could narrow the review’s outcomes. Considering the fact that the present review included only studies written in the English language, publication bias is plausible. Accordingly, further studies estimating the shear bond strength of denture teeth attached to CAD/CAM (milled) denture base resins and other new materials are necessary. Standardization of testing techniques in the literature would be useful for easier comparison between different studies.

## 5. Conclusions

Based on the present systematic review, the shear bond strength values between CAD/CAM (milled) denture base resins and artificial teeth are comparable or higher than the shear bond strength values between heat-polymerized acrylics and artificial teeth. In other words, bonding agents provide bonding strength that is at least similar to the conventional methods (compression molding technique).

It would be beneficial to use a larger number of specimens with standardized dimensions and a blinded testing machine operator in future studies to reduce the risk of bias.

## Figures and Tables

**Table 1:** Summary of the studies included in the systematic review.

Authors, Year	Title	n	Denture Base Material	Denture Teeth Material	Results (MPa)	Conclusions
Han et al. [45] (2020)	Shear bond strength between CAD/CAM denture base resin and denture artificial teeth when bonded with resin cement	10 per group	CAD/CAM (milled) (PMMA Block-pink prepolymerized resin block)	Highly cross-linked acrylic resin teeth (VITA MFT)	19.61 ± 3.07	Shear bond strengths of CAD/CAM denture base materials and resin denture teeth using resin cement are comparable to those of conventional methods
			CAD/CAM (milled) (PMMA Block-pink prepolymerized resin block	Composite resin teeth (Duracross Physio)	21.80 ± 3.00	
			CAD/CAM (milled) (PMMA Block-pink prepolymerized resin block	Composite resin teeth (Endura Posterio)	16.90 ± 3.48	
			CAD/CAM (milled) (Vipi Block-Pink)	Highly cross-linked acrylic resin teeth (VITA MFT)	19.79 ± 2.41	
			CAD/CAM (milled) (Vipi Block-Pink)	Composite resin teeth (Duracross Physio)	14.35 ± 4.56	
			CAD/CAM (milled) (Vipi Block-Pink)	Composite resin teeth (Endura Posterio)	17.20 ± 3.46	
			Heat-polymerized (Vertex Rapid Simplified)	Highly cross-linked acrylic resin teeth (VITA MFT)	18.84 ± 4.38	
			Heat-polymerized (Vertex Rapid Simplified)	Composite resin teeth (Duracross Physio)	19.31 ± 5.16	
			Heat-polymerized (Vertex Rapid Simplified)	Composite resin teeth (Endura Posterio)	10.17 ± 4.34	
Prpić et al. [29] (2020)	Comparison of shear bond strengths of different types of denture teeth to different denture base resins	8 per group	CAD/CAM (milled) (IvoBase CAD)	Acrylic teeth (SR Orthotyp S PE)	12.56 ± 2.92	Shear bond strength values between CAD/CAM (milled) denture base resins and different types of prefabricated teeth showed high shear bond strength values and are comparable with conventional methods
			CAD/CAM (milled) (IvoBase CAD)	Nanohybrid composite teeth (Phonares II Typ)	15.04 ± 1.68	
			CAD/CAM (milled) (IvoBase CAD)	Cross-linked teeth (SR Orthotyp DCL)	12.84 ± 3.21	
			CAD/CAM (milled) (IvoBase CAD)	CAD/CAM (milled) denture teeth (SR Vivodent CAD)	13.66 ± 4.27	
			Heat-polymerized (ProBase Hot)	Acrylic teeth (SR Orthotyp S PE)	18.10 ± 2.68	
			Heat-polymerized (ProBase Hot)	Nanohybrid composite teeth (Phonares II Typ)	12.81 ± 3.91	
			Heat-polymerized (ProBase Hot)	Cross-linked teeth (SR Orthotyp DCL)	14.29 ± 4.27	
Helal et al. [4] (2021)	Comparative effect of different surface treatments on the shear bond strength of two types of artificial teeth bonded to two types of denture base resins	10 per group	CAD/CAM (milled) (AvaDent PMMA Pucks)	Acrylic teeth (Acrostone)	9.64 ± 0.63	According to the results, there were significant differences in the SBS between the denture teeth bonded to heat-polymerized and CAD/CAM DBRs
			CAD/CAM (milled) (AvaDent PMMA Pucks)	Composite teeth (Eraylar- ostim)	7.92 ± 0.61	
			Heat-polymerized (Acrostone)	Acrylic teeth (Acrostone)	4.65 ± 0.54	
			Heat-polymerized (Acrostone)	Composite teeth (Eraylar- ostim)	3.28 ± 0.92	

**Table 2:** Risks of bias in the studies included in the systematic review.

Authors, Year	Samples Obtained through a Standardized Process	Single Operator of the Machine	Sample Size Calculation	Blinding of the Testing Machine Operator	Specimens, Test, and Formulas According to Standard Specifications	Risk of Bias
Han et al. [45] (2020)	0	2	2	2	0	Moderate
Prpić et al. [29] (2020)	0	2	2	2	0	Moderate
Helal et. al. [4] (2021)	0	2	2	2	0	Moderate

## Data Availability

Not applicable.
